# Local control of locally advanced uterine sarcoma achieved by cervical brachytherapy with gel spacer following cyclic hypofractionated radiation therapy (QUAD shot) in a patient who refused surgery

**DOI:** 10.1093/bjrcr/uaaf061

**Published:** 2025-12-05

**Authors:** Yasuo Kosugi, Naoya Murakami, Tatsuki Karino, Yoichi Muramoto, Terufumi Kawamoto, Masaki Oshima, Noriyuki Okonogi, Anneyuko I Saito, Jun Takatsu, Tatsuya Inoue, Kotaro Iijima, Akane Hashizume, Shigeki Tomita, Masaya Kato, Takafumi Ujihira, Shintaro Makino, Takashi Hirayama, Kazunari Fujino, Yasuhisa Terao, Naoto Shikama

**Affiliations:** Department of Radiation Oncology, Juntendo University Graduate School of Medicine, Tokyo, 113-8421, Japan; Department of Radiation Oncology, Juntendo University Urayasu Hospital, Chiba, 279-0021, Japan; Department of Radiation Oncology, Juntendo University Graduate School of Medicine, Tokyo, 113-8421, Japan; Department of Radiation Oncology, Juntendo University Graduate School of Medicine, Tokyo, 113-8421, Japan; Department of Radiation Oncology, Juntendo University Graduate School of Medicine, Tokyo, 113-8421, Japan; Department of Radiation Oncology, Juntendo University Graduate School of Medicine, Tokyo, 113-8421, Japan; Department of Radiation Oncology, Juntendo University Graduate School of Medicine, Tokyo, 113-8421, Japan; Department of Radiation Oncology, Juntendo University Graduate School of Medicine, Tokyo, 113-8421, Japan; Department of Radiation Oncology, Juntendo University Graduate School of Medicine, Tokyo, 113-8421, Japan; Department of Radiation Oncology, Juntendo University Urayasu Hospital, Chiba, 279-0021, Japan; Department of Radiation Oncology, Juntendo University Graduate School of Medicine, Tokyo, 113-8421, Japan; Department of Radiation Oncology, Juntendo University Graduate School of Medicine, Tokyo, 113-8421, Japan; Department of Radiation Oncology, Juntendo University Urayasu Hospital, Chiba, 279-0021, Japan; Department of Radiation Oncology, Juntendo University Graduate School of Medicine, Tokyo, 113-8421, Japan; Department of Pathology, Juntendo University Urayasu Hospital, Chiba, 279-0021, Japan; Department of Pathology, Juntendo University Urayasu Hospital, Chiba, 279-0021, Japan; Department of Pathology, Kasukabe Medical Center, Saitama, 344-8588, Japan; Department of Obstetrics and Gynecology, Juntendo University Urayasu Hospital, Chiba, 279-0021, Japan; Department of Obstetrics and Gynecology, Juntendo University Urayasu Hospital, Chiba, 279-0021, Japan; Department of Obstetrics and Gynecology, Juntendo University Urayasu Hospital, Chiba, 279-0021, Japan; Department of Obstetrics and Gynecology, Juntendo University, Tokyo, 113-8421, Japan; Department of Obstetrics and Gynecology, Juntendo University, Tokyo, 113-8421, Japan; Department of Obstetrics and Gynecology, Juntendo University, Tokyo, 113-8421, Japan; Department of Radiation Oncology, Juntendo University Graduate School of Medicine, Tokyo, 113-8421, Japan

**Keywords:** uterine sarcoma, external beam radiation therapy, QUAD shot regimen, intracavitary and interstitial (IC/IS) brachytherapy, gel spacer, definitive radiation therapy

## Abstract

Radiation therapy is not mentioned in various guidelines for primary treatment of uterine sarcoma. Radiation therapy is considered useful for palliative purposes, but an optimal radiation schedule has not been established. An 85-year-old woman was recommended to undergo surgery for locally advanced uterine sarcoma of unknown histological subtype, but she refused because of her poor physical and social circumstances. She instead received chemotherapy; however, the lesion progressed, and the patient opted for best supportive care. Palliative radiation therapy was indicated for genital bleeding. She thereafter received the QUAD shot regimen, in which 3.7 Gy was administered twice a day for 2 consecutive days and repeated every 4 weeks for a total dose of 44.4 Gy in 3 cycles. The lesion significantly decreased in size, and no new regional or distant metastases were identified. Although only palliative radiation therapy was initially intended, the favourable response with no new lesion development prompted us to offer additional brachytherapy for curative intent, and the patient accepted. She underwent 2 sessions of uterine cervical intracavitary and interstitial brachytherapy using a gel spacer, and local control of the lesion was achieved at 1-year and 3 months follow-up of treatment without major adverse events. In select patients for whom a favourable response is achieved after the QUAD shot, the addition of intracavitary and interstitial brachytherapy with a gel spacer may result in curative treatment. This therapy could be a very promising and attractive option for patients with uterine sarcoma who have complications or special social circumstances.

## Introduction

Uterine sarcoma (US) is an uncommon and heterogeneous malignant mesenchymal neoplasm, accounting for approximately 1% of all female genital tract malignancies and 3% to 7% of uterine cancers.^1^ The primary US histotypes are leiomyosarcoma, low-grade endometrial stromal sarcoma, high-grade endometrial stromal sarcoma, and undifferentiated endometrial sarcoma according to the most recent World Health Organization classification.^2^ Carcinosarcoma (CS), previously classified under the category of US until the 2000s, is now classified as a high-grade epithelial tumour because of its malignant epithelial component.^3^ Despite this reclassification, various retrospective studies on US and certain guidelines still incorporate CS alongside other subtypes. The diagnosis of US is typically established after surgery, although ultrasound and magnetic resonance imaging (MRI) may raise suspicion of a mesenchymal malignant tumour, particularly in patients with a rapidly expanding uterine mass. To ensure accurate assessment, pathological examinations should be performed at specialized centres with established expertise in this domain.^4^ The recommendations in the US guidelines are based on total hysterectomy with or without bilateral salpingo-oophorectomy as primary treatment, with chemotherapy and radiation therapy sometimes recommended after surgery.^5^ However, radiation therapy is not recommended as primary treatment except for patients with medical contraindications to surgery. Additionally, some guidelines recommend radiation therapy as palliative treatment, but the optimal schedule for radiation therapy has not been established.^4^

We herein describe a patient with US who refused surgery and successfully underwent a cyclic hypofractionated radiation therapy regimen (QUAD shot) as palliative radiotherapy. This treatment was followed by uterine cervical intracavitary and interstitial (IC/IS) brachytherapy using a gel spacer, achieving local control without major adverse events.

## Case

**Figure 1.**
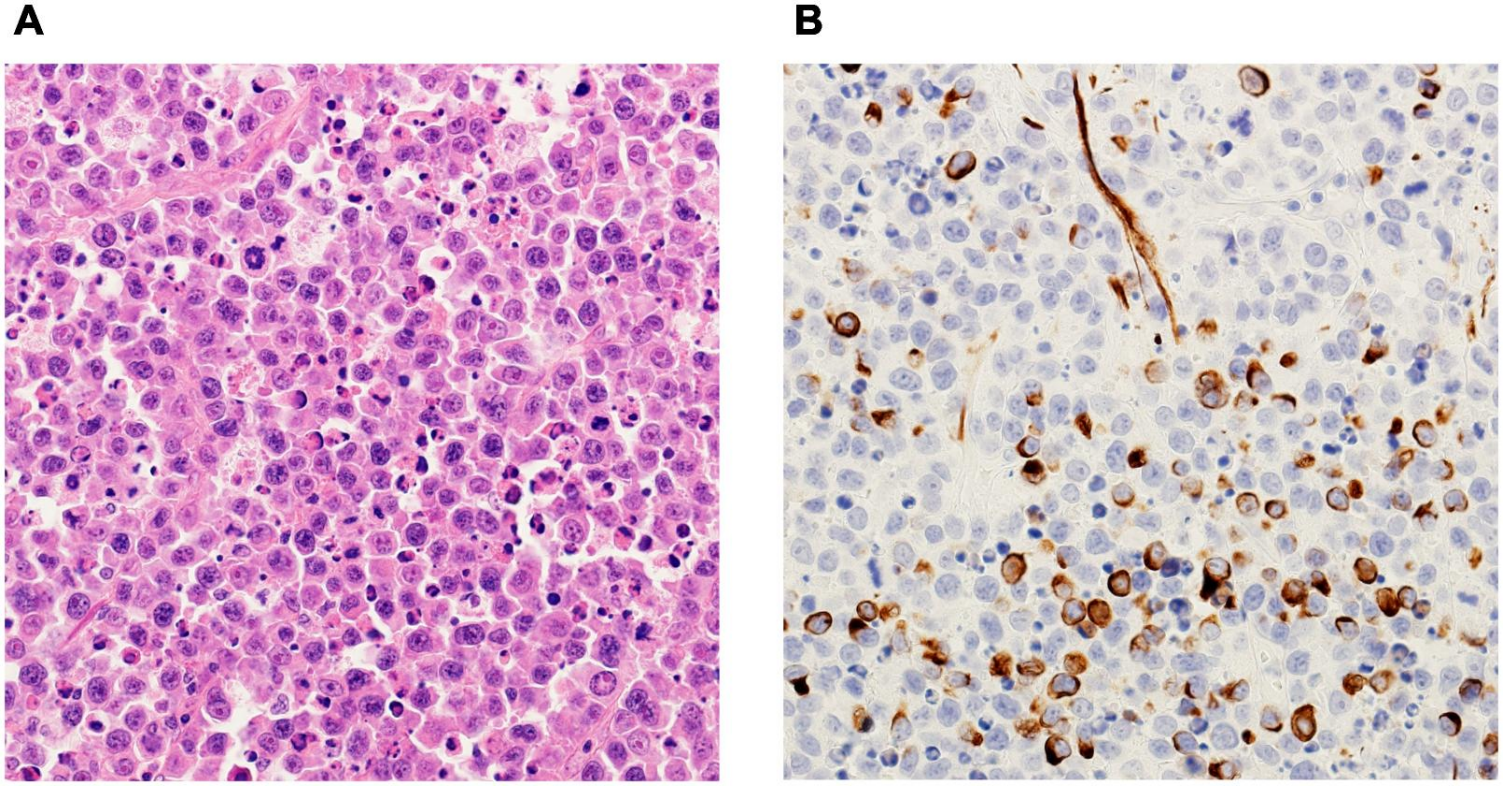
Pathological findings. (A) Haematoxylin-eosin staining shows that the tumour consisted of proliferating round cells. (B) Desmin staining shows that the tumour was focally positive.

An 85-year-old woman with type 2 diabetes mellitus, hypertension, and hyperlipidaemia visited our department of gynaecology in August 2022 for a genital bleeding episode, and a 40-mm cervical mass was noted. At the initial visit, her blood glucose level was 161 mg/dL and glycated haemoglobin (HbA1c) was 8.4%. She had been receiving insulin therapy for diabetes, an angiotensin II receptor blocker (telmisartan) for hypertension, and a statin (pravastatin) for hyperlipidaemia. Histological examination of the mass did not show an epithelial component, and additional immunostaining led to the diagnosis of sarcoma with differentiation into muscle tissue (Figure 1). Immunostaining revealed positivity for vimentin, desmin (focal), and Ki-67 (high) and negativity for cytokeratin AE1/3, LCA, CD56, S-100, Melan-A, α-smooth muscle actin, CD10, CD34, CD68, and oestrogen receptor. The myoglobin result was undetermined.

**Figure 2.**
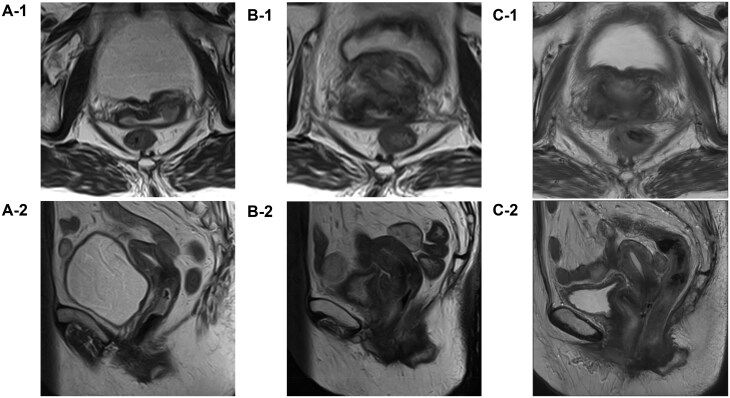
T2-weighted magnetic resonance imaging (A) before treatment, (B) after 3 courses of the QUAD shot, and (C) 6 months after completion of 2 courses of brachytherapy. (A-1, B-1, C-1) Axial and (A-2, B-2, C-2) sagittal plane images are shown. (A-1) Rupture of the cervical stromal ring in the left parametrium. (A-2) Vaginal extension of the cervical mass. (B) The vaginal extension is no longer evident, but the cervical mass appears to be partially residual. (C) The cervical mass is no longer visible.

**Figure 3.**
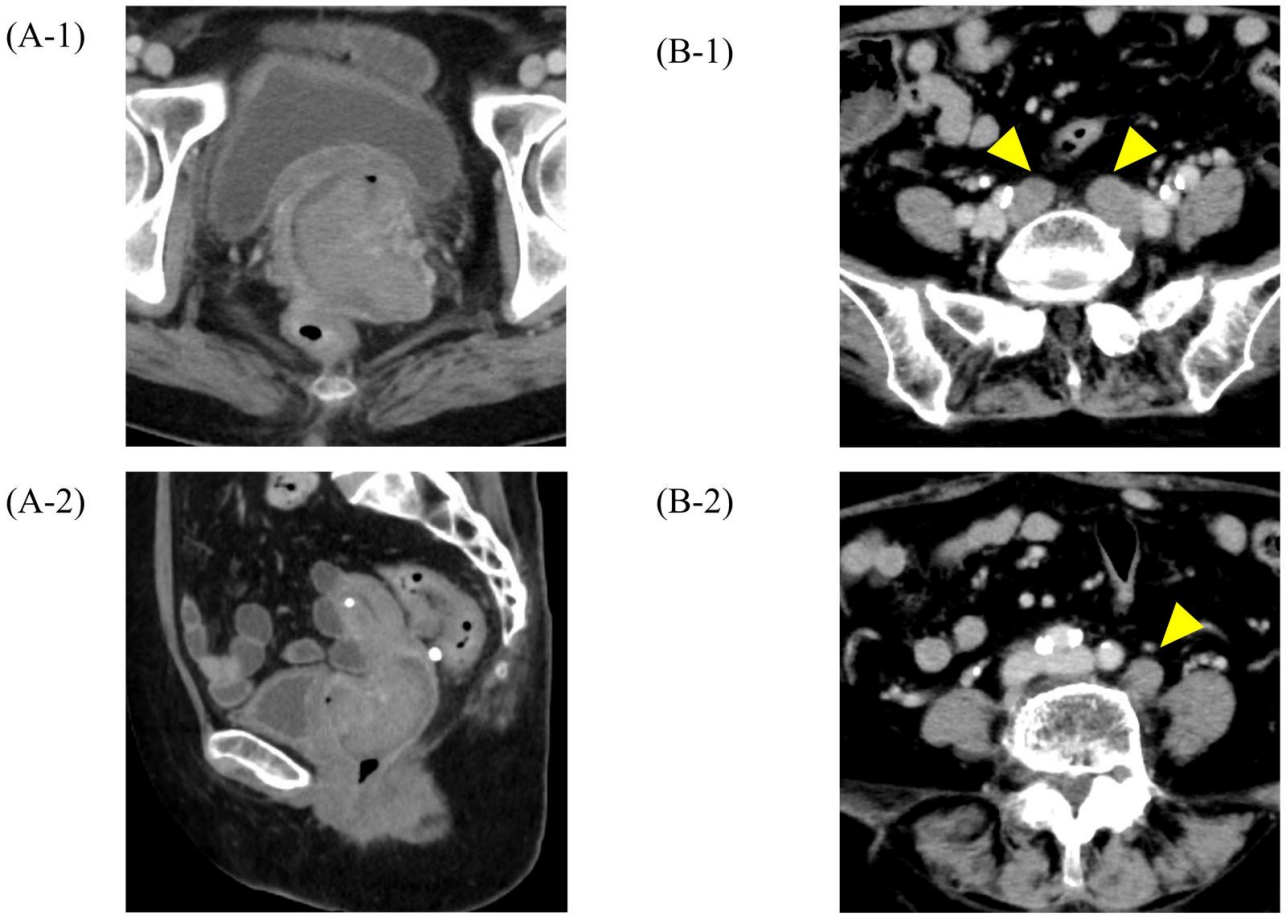
Contrast-enhanced CT images after 2 cycles of chemotherapy. (A-1) Axial and (A-2) sagittal plane images. The cervical mass had rapidly increased to 75 mm in size. (B-1) Axial CT image at the pelvic level and (B-2) at the para-aortic level taken 7 months after the completion of brachytherapy, showing recurrent lymph node metastases (arrowheads). These recurrent lesions were located outside the previously irradiated field.

Gynaecological examination revealed that the mass extended to the cranial third of the vagina. Pelvic contrast-enhanced MRI showed a cervical mass with stromal ring rupture, with direct invasion into the upper vagina and bilateral parametrium, but no evidence of invasion into the vesicovaginal septum, rectovaginal septum, or mesorectum (Figure 2A). Chest-to-pelvis CT showed no obvious regional or distant metastases. The diagnosis of locally advanced US was made based on the above findings, and the gynaecologist recommended total hysterectomy and bilateral adnexectomy. However, the patient refused surgery and referral to a distant heavy ion therapy centre due to her multiple comorbidities, advanced age, compromised physical condition, and social circumstances, including the need to care for her 90-year-old husband. To alleviate her symptoms, 2 courses of single-agent doxorubicin chemotherapy (75 mg/m^2^) were administered from November to December 2022. In January 2023, a CT scan for follow-up evaluation showed that the cervical mass had rapidly increased to 75 mm (Figure 3A). Chemotherapy was discontinued, and the patient transitioned to best supportive care. As part of her palliative care, she considered the introduction of home nursing care and a palliative care hospital. However, she developed genital bleeding that required a blood transfusion. She was referred to our department of radiation oncology for palliative radiation therapy to control the bleeding in March 2023.

**Figure 4.**
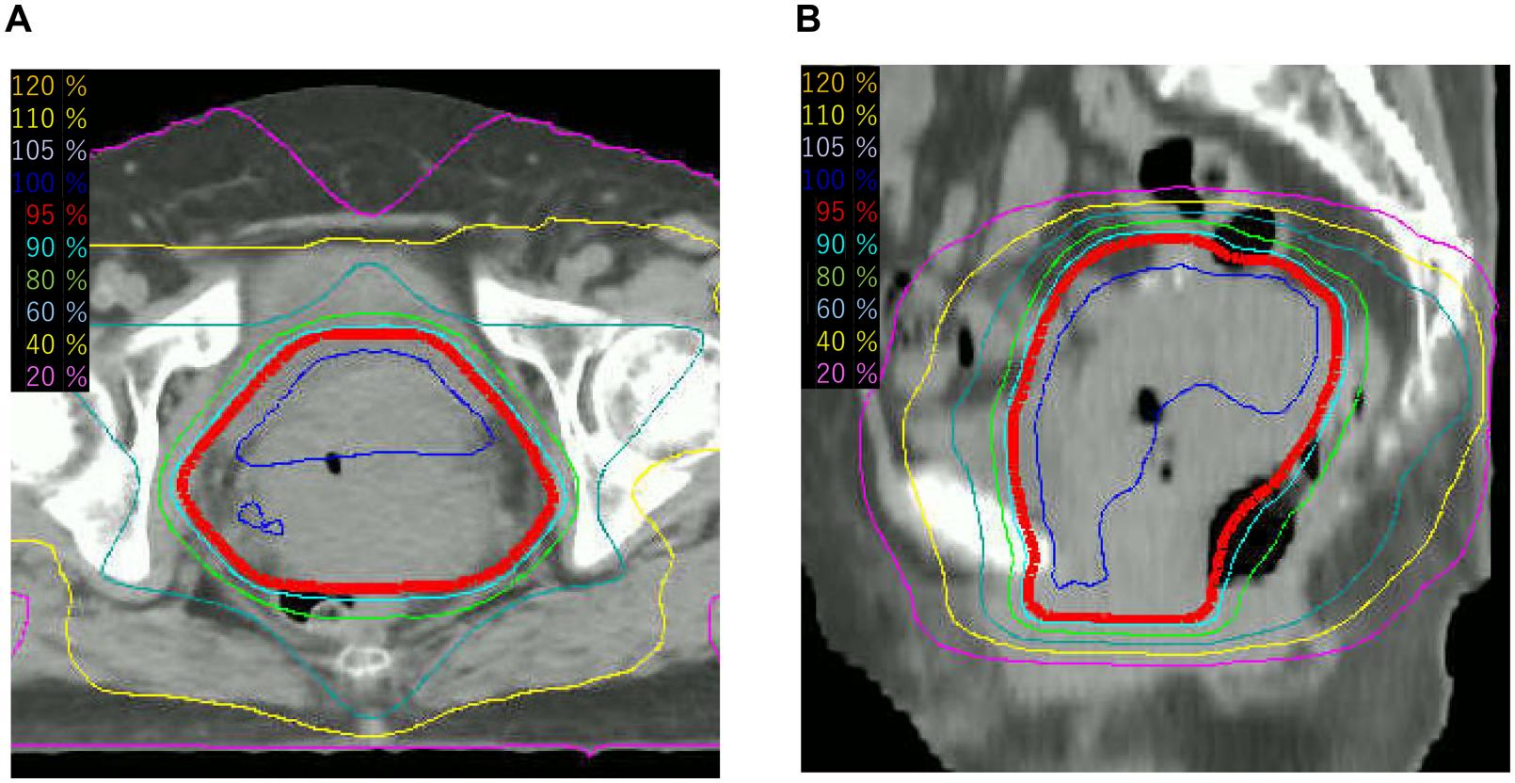
Isodose curves for the QUAD shot. (A) Axial view. (B) Sagittal view.

At the initial consultation in the radiation oncology department, the treatment plan was reconfirmed with the patient, and she expressed her wish to receive palliative radiation therapy, consistent with her earlier emphasis on social circumstances. Therefore, no additional imaging studies such as MRI were performed, in accordance with the patient’s wishes, and palliative radiation therapy was planned accordingly. Although the optimal schedule of palliative radiotherapy for US has not been established, we considered that the patient’s physical status (Eastern Cooperative Oncology Group performance status of 1) and social situation did not allow for consecutive daily visits to receive radiation therapy. Therefore, the QUAD shot was adopted. The first course (14.8 Gy in 4 fractions over the course of 2 days) was performed with 3D conformal radiation therapy in April 2023 (Figure 4). The clinical target volume (CTV) was set over the uterus and adnexa and the entire vagina, and the planning target volume margins were extended 1.5 cm in all directions and irradiated from 6 directions using 10-MV X-rays from a linear accelerator (Elekta Synergy Platform; Elekta Instrument AB, Stockholm, Sweden). For each external radiation, 350 mL of drinking water and 1 hour of urine storage were used to minimize the inter-fraction movement of the uterus and reduce the unneeded irradiation to the bowel. Daily image guidance was performed using linacgraphy (LG) with the same linear accelerator, as cone-beam CT (CBCT) was not available on this system. Follow-up CT 1 month after the first QUAD shot course showed good reduction of the cervical mass; therefore, a total of 3 courses of the QUAD shot, delivering 44.4 Gy in total, were irradiated every 4 weeks until June X + 1.

**Figure 5.**
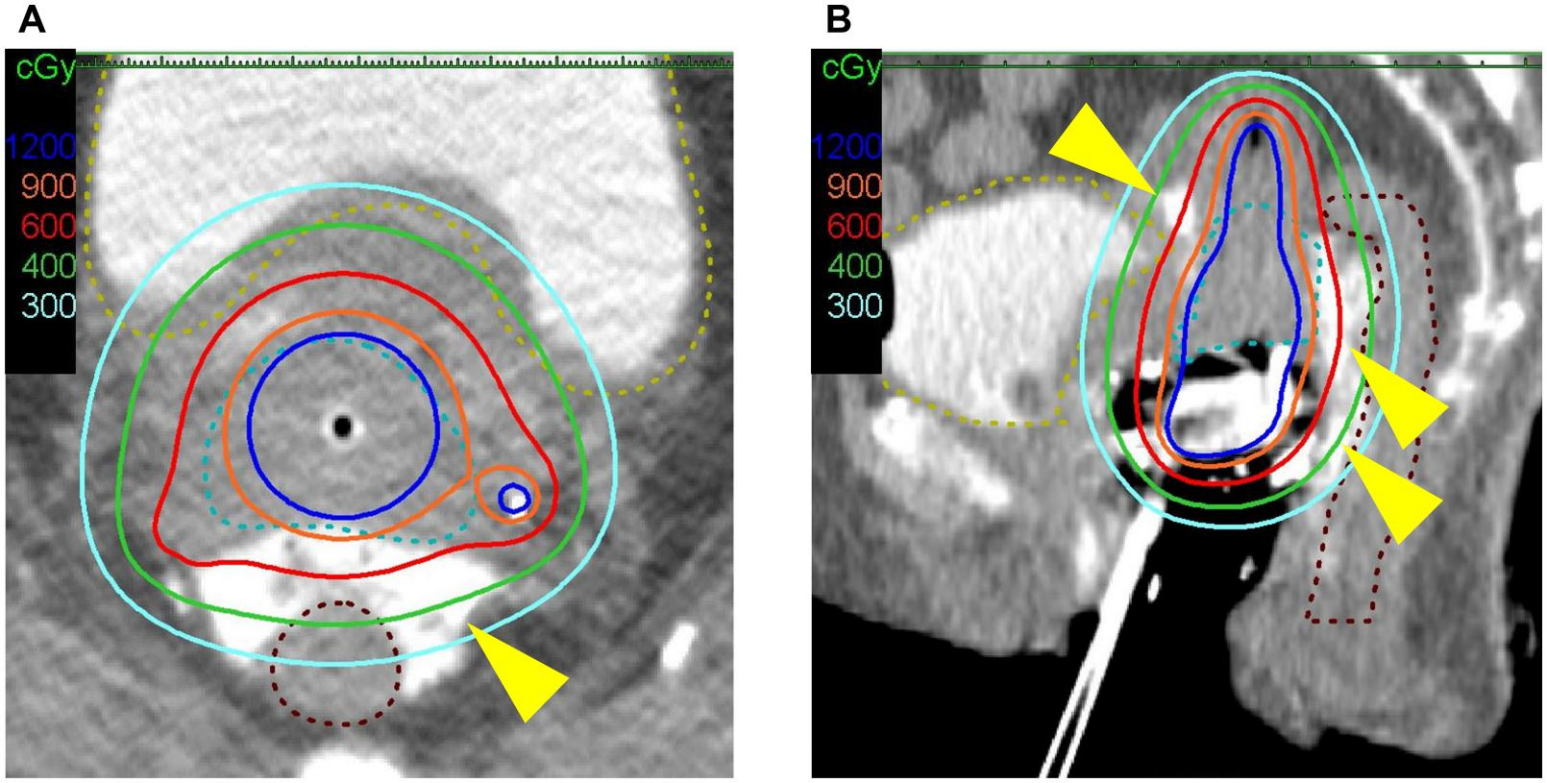
Isodose curves for cervical uterine brachytherapy using a gel spacer. (A) Axial view. (B) Sagittal view. Yellow dashed line: bladder. Brown dashed line: rectum. Light blue dashed line: high-risk clinical target volume. A gel spacer (MucoUp^®^; arrowhead) was inserted between the anterior vaginal wall and the bladder (vesicovaginal septum) and between the posterior wall of the vagina and the rectum (rectovaginal septum) with transrectal ultrasound guidance. After spacer insertion, the distance between the CTV_HR_ and the rectum was 9-12 mm on treatment planning CT. The MucoUp prevents the 6-Gy isodose line from crossing the contours of the bladder and rectum.

An MRI scan in June X + 1, 2 weeks after the 3 courses, showed that the mass had decreased to 35 mm in size, and contrast-enhanced CT from the chest to the pelvis showed no new regional or distant metastases (Figure 2B). Although the 3 courses of the QUAD shot had achieved a haemostatic effect, which was the initial goal of the palliative radiation therapy, the tumour had also shown an unexpectedly favourable response. Therefore, we offered the patient additional brachytherapy with potential curative intent. However, she requested further radical radiotherapy. Because radical radiotherapy for US is not a typical treatment, the patient was given a full explanation of the risk of severe radiation-related sequalae, such as bowel perforation or bladder bleeding. Additional uterine cervical brachytherapy was then scheduled with her consent. The combination of IC/IS brachytherapy was performed twice in July 2023 using a gel spacer to reduce the dose to organs at risk (OARs) while increasing higher dose per fraction to the tumour (Figure 5).^6^^,^^7^ High-dose-rate brachytherapy was performed with an Ir-192 remote afterloading system (microSelectron v3; Elekta, Stockholm, Sweden). Brachytherapy procedure was performed using intravenous hydroxyzine hydrochloride following the caudal block, midazolam, and fentanyl to obtain adequate pain relief and sedation. To enhance dose delivery while sparing the rectum and bladder, a hyaluronic acid gel spacer (MucoUp; Seikagaku Co., Tokyo, Japan) was inserted under transrectal ultrasound guidance between the anterior vaginal wall and the bladder (vesicovaginal septum) and between the posterior vaginal wall and the rectum (rectovaginal septum). The procedure involved hydrodissection with saline followed by injection of MucoUp, which was mixed with contrast agent to enable CT visualization. This technique is described in further detail in a prior report.^7^ Each session used a tandem and ovoid applicator in combination with 2 additional interstitial needles inserted perineally. CT images (2-mm slice thickness) were acquired in the treatment room using an Aquilion LB scanner (Canon Medical Systems Corp., Tochigi, Japan) with the patient in the lithotomy position. Contouring of the high-risk clinical target volume and OARs was performed with reference to guidelines for cervical cancer brachytherapy,^8^^,^^9^ and treatment planning was carried out using the Oncentra Brachy v4.6 system (Elekta, Stockholm, Sweden). Dose calculation was performed based on the Manchester method, delivering the prescribed dose to the A point. Subsequently, 10%-20% of dwell weight was added for the additional interstitial needles, followed by manual modification to meet the dose constraints for the CTV_HR_ and OARs. The combined dose of external beam radiation therapy and brachytherapy for the OARs and target volume is shown in Table 1.

**Table 1.** Combined dose of EBRT and brachytherapy for organs at risk and target volume (CTV_HR_ D90%).

	α/β	EQD_2_ (Gy)
**CTV_HR_ D90%**	10	75.1
	4	89.7
**Bladder D2cm^3^**	3	71.1
**Rectum D2cm^3^**	3	70.8
**Small bowel D2cm^3^**	3	63.4
**Sigmoid D2cm^3^**	3	64.2

The dosimetric evaluation was performed by summing the CTV_HR_ D90% or the D2cm^3^ of organs at risk for each session with EBRT and brachytherapy. The EBRT and brachytherapy doses were converted to EQD_2_. EQD2 for CTV_HR_ was calculated using α/β = 4 Gy (sarcoma), with α/β = 10 Gy (cervical cancer) shown for comparison. Values for organs at risk were calculated using α/β = 3 Gy. The following formula was used to calculate the EQD_2_: EQD_2_ = *D* × ((*d* + α/β)/(2+α/β)), where *D* is the total dose and *d* is the dose per fraction.

Abbreviations: CTV_HR_ D90%, minimum dose delivered to 90% of the high-risk clinical target volume, D2cm^3^, minimum dose delivered to the highest irradiated 2 cm^3^ volume; EBRT, external beam radiation therapy; EQD_2_, biological equivalent dose in 2 Gy.

The tumour responded well to radiation treatment, and a gynaecological examination 3 months after treatment showed no mass on transvaginal ultrasound and no malignant cells in the cytology tissue. No new metastases were identified in a cervical-to-pelvic contrast-enhanced CT scan.

Immediately after brachytherapy, the patient developed an acute adverse event of grade 1 cystitis according to the Common Terminology Criteria for Adverse Events version 5; however, the cystitis improved 1 month after treatment. No symptoms of treatment-induced late adverse events were subsequently observed. The patient was free of recurrence or metastasis on follow-up gynaecological examinations, and pelvic contrast-enhanced MRI at 6 months post-treatment (Figure 2C). Seven months after the completion of IC/IS brachytherapy, a contrast-enhanced CT scan from the cervical to pelvic regions revealed recurrent metastases in the pelvic and para-aortic lymph nodes (Figure 3B). Consequently, the patient underwent 3 additional courses of the QUAD shot regimen. A follow-up CT scan performed 1 year and 3 months after the initial treatment (6 months after the additional treatment) showed no recurrence or metastasis, including the primary tumour and lymph nodes.

## Discussion

US is a rare and heterogeneous disease. The role of radiotherapy in the management of US is controversial in international guidelines, probably because of the lack of strong evidence.^4^ The tumour stage is the single most important prognostic factor.^1^ In 2009, a new FIGO staging system was developed for US. The revised staging system has 2 divisions: 1 for leiomyosarcoma and endometrial stromal sarcoma and 1 for adenosarcoma. CS is now classified using the endometrial carcinoma staging system.^10^ Our patient’s tumour would have been classified as FIGO stage IIIB if it had been CS and stage IIB otherwise. In this case, the histological subtype of uterine sarcoma could not be determined due to limitations of the biopsy specimen. Although CS or adenosarcoma were suspected, based on the patient’s advanced age, further pathological confirmation was not possible. Adenosarcoma is considered a tumour with a good prognosis. In cases with invasion of the muscle layer, however, as in the present case, the prognosis is considered poor. CS also generally has a poorer prognosis than grade 3 endometrial carcinoma. The rapid tumour growth after chemotherapy in our patient also suggested that she still had sarcoma with a poor prognosis.^1^ Palliative radiation therapy is a recognized modality for alleviating symptoms in individuals with advanced or metastatic cancer. However, a standardized regimen for palliative irradiation in patients with gynaecologic malignancies has not yet been established.^11^ The QUAD shot, a cyclical hypofractionated radiation therapy initially applied to advanced pelvic malignancies in palliative settings,^12^ has recently demonstrated successful application in palliative care for head and neck cancers.^13^ Administered over 2 consecutive days per QUAD shot, this approach offers flexibility for subsequent sessions and is particularly beneficial for patients with a compromised general condition. A practical 4-week interval between QUAD shots allows normal tissues to recover from mild (grade 1-2) radiation toxicities, thereby minimizing the risk of developing severe (grade ≥3) toxicities.^14^ In the current case, the patient experienced favourable tumour shrinkage and haemostasis with no significant radiation-related adverse events. The schedule of 2 days per course was compatible with her physical and social needs, and her acceptance of the treatment was good. In this case, 3D conformal radiotherapy was selected for external beam delivery. Although intensity-modulated radiation therapy could have potentially provided better dose conformity, at the time of treatment, palliative intensity-modulated radiation therapy was not reimbursed under the national health insurance system. Therefore, 3D conformal radiotherapy was used as the most feasible modality. After 3 courses of the QUAD shot, our patient felt that the treatment had been effective and requested to undergo radical radiotherapy. Therefore, we considered additional brachytherapy to increase the likelihood of curing the disease after ensuring that the patient understood the risks, although primary radiotherapy for US has been rarely reported to date, and its outcomes were very poor.^15^ The QUAD shot was originally developed with a maximum of 3 courses. Although palliative response rates are reportedly correlated with the number of cycles, this regimen is insufficient for long-term disease control.^13^ Some reports of 4 or more courses being conducted and dose calculations show that the biological effect is similar to that of a radical dose.^16^ In the present case, however, OARs such as the bowel and bladder were in close proximity to the target volume, and treatment with more than 4 courses appeared difficult. Therefore, we decided to minimize the dose to the OARs by IC/IS brachytherapy using a gel spacer, which was previously reported as the first-in-human application of MucoUp for cervical cancer brachytherapy. This hyaluronic acid compound was originally used to lift the submucosal tissue during endoscopic resection for superficial malignancies in the gastrointestinal tract.^6^

High-precision external radiation therapy such as intensity-modulated radiation therapy and stereotactic body radiotherapy were also considered in this case. However, high-precision external radiation therapy cannot replace brachytherapy in the treatment of cervical cancer because of its higher toxicity and lower survival rate compared with brachytherapy.^17^^,^^18^ Instead, IC/IS brachytherapy using a gel spacer was able to deliver the highest possible dose to the target volume while adhering to the dose constraints recommended by the Gynecological (GYN) GEC-ESTRO Working Group (Table 1); this was accomplished by the good dose distribution provided by IC/IS brachytherapy and the dose reduction to the OARs by the gel spacer.^8^ The total dose constraints for the OARs were 75 Gy to the rectum D2cm^3^, 90 Gy to the bladder D2cm^3^, 75 Gy to the sigmoid colon D2cm^3^, and 70 Gy to the small bowel D2cm^3^, expressed in equivalent dose in 2-Gy fractions (EQD2) with α/β = 3 Gy. Although no CT imaging was obtained prior to spacer insertion, it is estimated—based on the dose distribution—that the rectal D2cm^3^ EQD2 would have been approximately 81.1 Gy without the spacer, which would have exceeded the recommended constraint of 75 Gy for the rectum. The dose index of the target volume [D90% of high-risk CTV (CTV_HR_ D_90％_)] was 75.1 Gy with α/β = 10 in EQD_2_, which is below the ASTRO-recommended dose of 80 Gy for image-guided adaptive brachytherapy for cervical cancer.^9^ With this irradiation dose, a curative effect for US is unlikely. However, sarcomas have been reported to exhibit a relatively low α/β ratio, estimated at approximately 4 Gy, suggesting a higher sensitivity to hypofractionated regimens. In this case, the CTV_HR_ D90% calculated with an α/β of 4 Gy was 89.7 Gy,^19^ indicating a potentially effective biological dose. The high dose per fraction delivered by both the QUAD shot regimen and high-dose-rate IC/IS brachytherapy with a gel spacer might have offered a biological advantage in treating such radioresistant tumours. This approach is consistent with previous reports demonstrating the efficacy of high-dose-per-fraction regimens, including the QUAD shot, in tumours with low α/β ratios such as melanoma, urothelial carcinoma, thyroid carcinoma, and various sarcomas.^14^^,^^16^^,^^20^ These findings support the radiobiological rationale for selecting this treatment strategy in the present case.

Further follow-up is needed to confirm long-term local control and toxicity in this case. Because we experienced only 1 case in which US was successfully treated by the combination of the QUAD shot and IC/IS brachytherapy with gel spacer, it is unknown whether this strategy was particularly effective for this specific patient or if it can be widely applied to other patients with US. However, in the few reports of radical radiotherapy for US, most cases have local recurrence or death within 1 year,^15^ making this case a promising treatment option. The optimal dose fractionation for each of external radiation therapy and brachytherapy during radical radiotherapy is unknown. Based on the results of a previous study showing that response rate increase with 3 or more courses of QUAD shot when QUAD shot is used for palliative irradiation, IC/IS brachytherapy with gel spacer may be useful for delivering a curative dose to US while meeting OARs dose constraints,^8^ when implementing radical brachytherapy in selected cases after 3 courses of QUAD shot.

Therefore, we believe our case study warrants widespread dissemination within the medical community and will contribute to further accumulation of knowledge regarding this rare gynaecological malignancy. This may in turn improve the outcome of this potentially fatal disease.

## Conclusion

After 3 courses of the QUAD shot as palliative treatment for US, curative treatment may be offered by adding IC/IS brachytherapy with a gel spacer in select patients. These treatments could be very promising and attractive options for advanced-age patients with US who have complications or specific social circumstances. Further research into optimal case selection and irradiation schedules is necessary.

## Learning points

- The QUAD shot regimen provided effective palliative tumour control in an elderly patient with uterine sarcoma and comorbidities.
- In selected cases, favourable response to the QUAD shot may allow for the consideration of curative-intent treatment.
- IC/IS brachytherapy with a gel spacer enabled safe dose escalation while sparing organs at risk.
- The combination of the QUAD shot and high-dose-per-fraction brachytherapy may be biologically advantageous for radioresistant tumours like uterine sarcoma, which have a low α/β ratio.
- This case suggests that, in patients with uterine sarcoma initially considered for palliation, a favourable response to the QUAD shot could justify the addition of IC/IS brachytherapy with curative intent.

## Data Availability

Research data are stored in an institutional repository and will be shared upon request to the corresponding author.

## Generative AI and AI-assisted technologies in the writing process

During the preparation of this work, the authors used ChatGPT in order to improve readability and language. After using this tool, the authors reviewed and edited the content as needed and take full responsibility for the content of the publication.
